# Supplementary material for: Treemendous: an R package for integrating taxonomic information across backbones
Source: PeerJ. 2024 Feb 28;12:e16896. doi: 10.7717/peerj.16896 (PMC10908262; doi:10.7717/peerj.16896)
Supplement: Supplemental Information 1 [file peerj-12-16896-s001.pdf]

Package ‘treemendous’

August 10, 2023

Title An R package for standardizing taxonomic names of tree species

Version 1.1.1

Description
Treemendous is an open-source software package for the R programming environment that provides a toolset for standardizing tree species names according to four publicly available backbones: World Flora Online (WFO), the Botanical Gardens Convention International (BGCI), the World Consensus on Vascular Plants (WCVP) and the Global Biodiversity Information Facility (GBIF). The package simultaneously leverages information and relationships across all these backbones to increase matching rates and minimize data loss, while ensuring the resulting species are accepted and consistent with a single reference backbone. The package provides a flexible workflow depending on the use case, in which users can chain together different functionalities ranging from simple matching to a single backbone, to graph-based iterative matching using synonym-accepted relations across all backbones in the database. In addition, the package allows users to `translate' one tree species list into another, streamlining the assimilation of new data into preexisting datasets or models.

License CC BY 4.0

Encoding UTF-8

Roxygen list(markdown = TRUE)

RoxygenNote 7.2.3

Imports assertthat,
dplyr,
fuzzyjoin,
igraph,
magrittr,
Matrix,
memoise,
progress,
purrr,
readr,
stats,
stringr,
tibble,
tidyr

Depends R (>= 3.6)

LazyData true

LazyDataCompression xz

Suggests testthat (>= 3.0.0)

Config/testthat/edition 3

R topics documented:

direct_match 2
direct_match_species_within_genus 3
enforce_matching 3
fia 5
fuzzy_match_genus 5
fuzzy_match_species_within_genus 6
genus_match 7
highlight_flags 7
iucn 9
matching 10
resolve_synonyms 11
sequential_matching 12
suffix_match_species_within_genus 12
summarize_output 13
translate_trees 14
Treemendous.Trees 14

Index **17**

`direct_match` *Direct Match Species & Genus Binomial*

Description

Tries to directly match Genus + Species Binomial to `Treemendous.Trees`.

Usage

```
direct_match(df, backbone = NULL, target_df = NULL)
```

Arguments

`df`	`tibble` containing the species binomial split into the columns `Orig.Genus` and `Orig.Species`.
`backbone`	specifies which backbone is used: needs to be a subset of `c('BGCI', 'WCVP', 'WFO', 'GBIF')` or `NULL` if the whole database should be used.
`target_df`	is used if the user wants to provide a custom target dataset. The parameter is intended only for compatibility with the function translate_trees and not for direct usage.

Value

Returns a `tibble` with the additional logical column `direct_match`, indicating whether the binomial was successfully matched (TRUE) or not (FALSE)

Examples

```
iucn %>% direct_match()
```

`direct_match_species_within_genus` *Direct Match Species within Genus*

Description

Tries to directly match the specific epithet within an already matched genus in `Treemendous.Trees`

Usage

```
direct_match_species_within_genus(df, backbone = NULL, target_df = NULL)
```

Arguments

`df`	`tibble` containing the species binomial split into the columns `Orig.Genus` and `Orig.Species`.
`backbone`	specifies which backbone is used: needs to be a subset of `c('BGCI', 'WCVP', 'WFO', 'GBIF')` or `NULL` if the whole database should be used.
`target_df`	is used if the user wants to provide a custom target dataset. The parameter is intended only for compatibility with the function translate_trees and should not be directly used.

Value

Returns a `tibble` with the additional logical column `direct_match_species_within_genus`, indicating whether the specific epithet was successfully matched within the matched genus (TRUE) or not (FALSE).

Examples

```
iucn %>% dplyr::mutate(Matched.Genus = Orig.Genus) %>% direct_match_species_within_genus()
```

`enforce_matching` *Enforce Matching for Unmatched Species According to a Specified Backbone*

Description

`enforce_matching()` can be called after `matching()`. The function tries to match all unmatched species, by making use of the synonym-accepted relations present in the backbones `WFO`, `WCVP` and `GBIF`. A graph connecting all synonyms with accepted species is created and used to look for matches at increasing distance in this graph according to the desired backbone.

Usage

```
enforce_matching(df, backbone, target_df = NULL, max_iter = 3)
```

Arguments

df
: tibble which is the output of matching() or sequential_matching() and therefor contains the columns Matched.Genus and Matched.Species. May contain additional columns, which will be ignored.

backbone
: specifies which backbone is used: needs to be one of c('BGCI', 'WCVP', 'WFO', 'GBIF').

target_df
: is used if the user wants to provide a custom target dataset. The parameter is intended only for compatibility with the function translate_trees and should not be directly used.

max_iter
: maximum distance (depth) in the graph for two species to be successfully enforce matched.

Details

This function is useful when you want to increase the proportion of matched species against a single target backbone. The package *igraph* is used to create an undirected graph g connecting all synonyms with accepted species according the databases WFO, WCVP and GBIF. Vertices represent species names, and edges represent synonym-accepted relations between two species according to at least one backbone. Additionally, two species names that can be matched via fuzzy-matching (maximum string-dist of two) are also connected with an edge. To find these, each species name is matched against the whole database (excluding its own name).

From the output of matching(), all unmatched species are matched to all three backbones via matching(c('WFO', 'WCVP', 'GBIF')). The functions checks vertices that are at most max_iter (default = 3) edges apart in the graph g. For multiple matches, the algorithm always selects the first match, i.e. the target vertex with lower ID_matched in Treemendous.Trees to ensure reproducibility. By default, the function allows a maximum depth of three steps to search for an match in the target backbone, with the output field enforced_matching_dist denoting the depth of the match for each species (1, 2, or 3). Filtering by this column allows the user to be more restrictive (depth $=1$), at the cost of incorrectly missing some matches, or be increasingly permissive with the matches (depth $=2$ or 3), at the cost of potentially lumping species together. Depending on the application, these different scenarios may be more or less preferable, and can be selected on a case-by-case basis.

Value

A tibble with matched species in Matched.Genus and Matched.Species. Along with the process information of matching(), the function returns the logical column enforced_matched, stating whether the species was successfully matched by enforce_matching(), and the distance in the neighborhood graph g.

Examples

```
output <- iucn %>% matching('BGCI') %>% enforce_matching('BGCI')
output %>% summarize_output()
```

fia — *Cleaned Master Tree Species list from FIA*

Description

A cleaned dataset containing trees the Forest Inventory and Analysis (FIA) program of the U.S. Forest Service. This dataset is used in the example usage section of the manuscript for the *Treemendous* package. The data was downloaded in November 2022 from the official webpage of the Forest Inventory and Analysis National Program and is available under the following link.

Usage

```
fia
```

Format

A data frame with 2171 rows and 2 variables:

Genus Genus name of species binomial

Species Specific epithet of the species binomial

fuzzy_match_genus — *Fuzzy Match Genus Name*

Description

Tries to fuzzy match the genus name to `Treemendous.Trees`. Uses `fuzzyjoin::stringdist()` to perform fuzzy matching.

Usage

```
fuzzy_match_genus(df, backbone = NULL, target_df = NULL)
```

Arguments

`df`	`tibble` containing the species binomial split into the columns `Orig.Genus` and `Orig.Species`.
`backbone`	specifies which backbone is used: needs to be a subset of `c('BGCI', 'WCVP', 'WFO', 'GBIF')` or `NULL` if the whole database should be used.
`target_df`	is used if the user wants to provide a custom target dataset. The parameter is intended only for compatibility with the function translate_trees and should not be directly used.

Value

Returns a `tibble` with the additional logical column `fuzzy_match_genus`, indicating whether the genus was successfully matched (TRUE) or not (FALSE). Further, the additional column `fuzzy_genus_dist` returns the distance for every match.

Examples

```
iucn %>%
    dplyr::mutate(Orig.Genus = stringr::str_replace(Orig.Genus, '.{1}$', '')) %>%
    fuzzy_match_genus()
```

---

fuzzy_match_species_within_genus
Fuzzy Match Species within Genus

Description

Tries to fuzzy match the species name to Treemendous.Trees within a genus. Uses fuzzyjoin::stringdist() to perform fuzzy matching.

Usage

```
fuzzy_match_species_within_genus(df, backbone = NULL, target_df = NULL)
```

Arguments

df	tibble containing the species binomial split into the columns Orig.Genus and Orig.Species.
backbone	specifies which backbone is used: needs to be a subset of c('BGCI', 'WCVP', 'WFO', 'GBIF') or NULL if the whole database should be used.
target_df	is used if the user wants to provide a custom target dataset. The parameter is intended only for compatibility with the function translate_trees and should not be directly used.

Value

Returns a tibble with the additional logical column fuzzy_match_species_within_genus, indicating whether the specific epithet was successfully fuzzy matched within the matched genus (TRUE) or not (FALSE).

Examples

```
iucn %>%
    dplyr::mutate(Orig.Genus = stringr::str_replace(Orig.Genus, '.{1}$', '')) %>%
    dplyr::mutate(Matched.Genus = Orig.Genus) %>%
    fuzzy_match_species_within_genus()
```

genus_match *Match Genus name*

Description

#' Tries to match the genus name to `Treemendous.Trees`.

Usage

```
genus_match(df, backbone = NULL, target_df = NULL)
```

Arguments

df	`tibble` containing the species binomial split into the columns `Orig.Genus` and `Orig.Species`.
backbone	specifies which backbone is used: needs to be a subset of `c('BGCI', 'WCVP', 'WFO', 'GBIF')` or `NULL` if the whole database should be used.
target_df	is used if the user wants to provide a custom target dataset. The parameter is intended only for compatibility with the function translate_trees and should not be directly used.

Value

Returns a `tibble` with the additional logical column `genus_match`, indicating whether the genus was successfully matched (TRUE) or not (FALSE)

Examples

```
iucn %>% genus_match()
```

highlight_flags *Highlight potential ambiguities introduced upon resolving synonyms to their accepted names*

Description

The user can call `highlight_flags()` from the output of `resolve_synonyms()` to investigate potential ambiguities when resolving synonyms to their accepted latin binomial names. These ambiguities can be of varying importance for different use-cases and should be carefully assessed for each use-case.

Usage

```
highlight_flags(df, backbone = NULL)
```

Arguments

df
: tibble which is the output of resolve_synonyms() and therefor contains the columns Accepted.Genus, Accepted.Species, Matched.Genus and Matched.Species. May contain additional columns, which will be discarded.

backbone
: specifies for which backbone(s) the flags should be appended. Needs to be one of (or a combination of) c('WCVP', 'WFO', 'GBIF') or NULL if all should be used.

Details

The following flags have been introduced in order to clarify and alleviate potential ambiguities that were introduced upon compilation of Treemendous.Trees. The flags always correspond to the latin binomial in the columns c(Matched.Genus, Matched.Species). The user is encouraged to check for their individual use-cases if she/he wants to exclude resolved names based on these flags. Below they are described in more detail.

The first column appended has the suffix _authorship_ambiguity and can be either TRUE or FALSE.

- TRUE: For a given latin binomial, there are multiple entries at taxonomic rank "Species" in the underlying backbone, which would be resolved to different latin binomials due to different authorships.
  - For instance, in the WCVP backbone, two entries are present for the latin binomial *Acer flabellatum* at rank "Species" (*Acer flabellatum* Greene, *Acer flabellatum* Rehder). The first is considered a synonym of *Acer macrophyllum* Pursh, while the latter is considered a synonym of *Acer campbellii subsp. flabellatum* (Rehder) A.E.Murray.
- FALSE: no ambiguity

The second column appended has the suffix _infraspecific_ambiguity and can be either TRUE or FALSE.

- TRUE: For a given latin binomial, there are multiple entries at different taxonomic ranks (Species, Subspecies, Variety or Form) in the underlying backbone, which would be resolved to different latin binomials.
  - For instance, in the WCVP backbone, four entries are present for the latin binomial *Nothofagus obliqua*, one at rank "Species" (*Nothofagus obliqua*), two at rank "Subspecies" (*Nothofagus obliqua subsp. andina* and *Nothofagus obliqua subsp. valdiviana*) and one at rank "Variety" (*Nothofagus obliqua var. macrocarpa*). While the former three would all be resolved to the latin binomial *Nothofagus obliqua*, the latter is considered to be a synonym of the accepted name *Nothofagus macrocarpa*. Resolve_synonyms() will however always resolve it to *Nothofagus obliqua*, because this was the only accepted name out of all the four entries. This flag should not be a problem if you are working with latin binomials from the beginning. However, if your initial species names consists of trinomials (infraspecific species names), then this flag can help you to identify ambiguous name resolving.
- FALSE: no ambiguity

The third column appended has the suffix _infraspecific_link and can be either TRUE or FALSE.

- TRUE: indicates the the resolved species was obtained by linking a trinomial to a corresponding Latin binomial. If an entry in a given backbone is pointing (meaning is a synonym of) to another entry which has been removed (in favor of another entry with the same latin binomial), then the linkage of the synonym-accepted relation was updated. If you are only interested in

resolving to the correct latin binomials, you can generally neglect this flag. However, be aware that the associated information of the resolved species (e.g. Authorship information) might not be correct in your case.

  - For instance, `WCVP` considers the species *Betula kwangsiensis* as a synonym of the accepted subspecies *Betula kweichowensis subsp. kweichowensis*. This subspecies however shares its latin binomial with the accepted species *Betula kweichowensis*. Therefore the species *Betula kwangsiensis* had to be relinked to the species *Betula kweichowensis* in order to resolve the latin binomials correctly.
  - Another example would be: According to `WFO`, the species *Abies shastensis* is a synonym of the accepted variety *Abies magnifica var. shastensis*. However, because there is also an accepted entry at rank species *Abies magnifica* (which is selected during the compilation of `Treemendous.Trees`), the species *Abies shastensis* has to be relinked to *Abies magnifica*.

- `FALSE`: no new linkaged was required

Value

The function outputs a `tibble` that includes the original species names, the matched species names, the accepted species names, and the corresponding flags for the matched species. It filters and returns only those entries where at least one flag was raised.

Examples

```
iucn_resolved <- iucn %>% matching('WCVP') %>% resolve_synonyms('WCVP')
iucn_resolved %>% highlight_flags('WCVP')
```

`iucn` *Selected Trees from the IUCN red list of Threatened Species*

Description

A dataset containing threatened tree species from the genus `Acer` and the families `Betulaceae`, `Nothofagaceae` and `Theaceae`. The data was downloaded in June 2022 from the official webpage of the International Union for Conservation of Nature (IUCN) and is available under the following link.

Usage

```
iucn
```

Format

A data frame with 384 rows and 2 variables:

Orig.Genus Genus name of species binomial

Orig.Species Specific epithet of the species binomial

matching — *Matches species names to* Treemendous.Trees

Description

This function takes species names and matches these against the internal database Treemendous.Trees. The function is a wrapper around the following functions:

- direct_match()
- genus_match()
- fuzzy_match_genus()
- direct_match_species_within_genus()
- suffix_match_species_within_genus()
- fuzzy_match_species_within_genus()

Usage

```
matching(df, backbone = NULL, target_df = NULL)
```

Arguments

df	tibble containing the species binomial split into the columns Genus and Species. May contain additional columns, which will be ignored.
backbone	specifies which backbone is used: needs to be a subset of c('BGCI', 'WCVP', 'WFO', 'GBIF') or NULL if the whole database should be used.
target_df	is used if the user wants to provide a custom target dataset. The parameter is intended only for compatibility with the function translate_trees and should not be directly used.

Details

First, direct_match() is called, which matches a name, when the exact same name (genus and specific epithet) is present in the database. If there was no direct match, genus_match() checks, whether the genus exists in the database. If the genus was not present, fuzzy_match_genus() is called, which tries to inexactly match genus names using the package *fuzzyjoin* based on an *optimal string alignment distance* of one, as implemented in *stringdist*. In addition to insertions, deletions and substitutions, the metric also considers transpositions (e.g. Quercus → Quecrus) as operations of distance one. If more than one genus matched, the alphabetically first match is picked, but the user is informed and encouraged to curate the ambiguous entries by hand. The maximal genus edit distance is set to one by design, because typos in genus names can be considered much rarer compared to the specific epithet and because genus names are usually quite short.

After the genus name has been matched, three functions are called within a certain genus. First, direct_match_species_within_genus() checks if the specific epithet is present in the matched genus. If not, suffix_match_species_within_genus() tries to capture gender-specific endings or other common suffixes. More specifically, the following suffixes are substituted c("a", "i", "is", "um", "us", "ae"). Next, the remaining unmatched species names are fuzzy matched with a maximal *optimal string alignment distance* of two.

The function matching() returns a tibble with the new columns Matched.Genus and Matched.Species containing the matched names, or NA if there was no match. Further, a logical column is added for

every function called to allow the user to inspect which functions were for every name during the process. When a process column shows `NA`, then this function was not called for the given name, because it was already matched with a preceding function.

Value

Returns a `tibble`, with the matched names in `Matched.Genus` and `Matched.Species`. Process information is added as individual columns for every function. The original input columns `Genus` and `Species` are renamed to `Orig.Species` and `Orig.Genus`.

Examples

```
iucn %>% matching()
```

---

resolve_synonyms — *Resolve Synonyms for Matched Species Names*

Description

This function is called after `matching()` and resolve synonyms based on the database `Trees.Full`. Information on synonyms comes from the databases `WCVP`, `WFO` and `GBIF`. `WFO` is considered to be the primary backbone, `WFO` the secondary, and `GBIF` the tertiary.

Usage

```
resolve_synonyms(df, backbones = NULL)
```

Arguments

`df`	: tibble containing the two columns `Matched.Genus` and `Matched.Species`, which need to be created by calling `matching()`.
`backbones`	specifies the order in which synonyms are resolved: needs to be a subset of c('BGCI', 'WCVP', 'WFO', 'GBIF') or NULL if the default ordering c('BGCI', 'WFO', 'WCVP', 'FIA', 'PM', 'GBIF') should be used .

Value

tibble with two new columns: `Accepted.Genus` and `Accepted.Species`

Examples

```
backbones = c('BGCI', 'WFO')
iucn %>% matching(backbones) %>% resolve_synonyms(backbones)
```

sequential_matching *Sequentially Matches Species Names to* Treemendous.Trees

Description

This function is a wrapper around `matching()`, which matches species names against the internal database `Treemendous.Trees` according to a specified backbone. `matching()` is called for every individual backbone provided via the argument `sequential_backbones` in the order of appearance.

Usage

```
sequential_matching(df, sequential_backbones)
```

Arguments

df	`tibble` containing the species binomial split into the columns `Genus` and `Species`. May contain additional columns, which will be ignored.
sequential_backbones	specifies the backbone which are sequentially used: needs to be a subset of `c('BGCI', 'WCVP', 'WFO', 'GBIF', 'FIA', 'PM')`.

Value

Returns a `tibble`, with the matched names in `Matched.Genus` and `Matched.Species`. Process information is added as individual columns for every function. The original input columns `Genus` and `Species` are renamed to `Orig.Species` and `Orig.Genus`.

Examples

```
iucn %>% sequential_matching(sequential_backbones = c('WFO', 'BGCI'))
```

suffix_match_species_within_genus *Suffix Match Species within Genus*

Description

Tries to match the specific epithet by exchanging common suffixes within an already matched genus in `Treemendous.Trees`. The following suffixes are captured: `c("a", "i", "is", "um", "us", "ae")`

Usage

```
suffix_match_species_within_genus(df, backbone = NULL, target_df = NULL)
```

Arguments

df	`tibble` containing the species binomial split into the columns `Orig.Genus` and `Orig.Species`.
backbone	specifies which backbone is used: needs to be a subset of `c('BGCI', 'WCVP', 'WFO', 'GBIF')` or `NULL` if the whole database should be used.
target_df	is used if the user wants to provide a custom target dataset. The parameter is intended only for compatibility with the function translate_trees and should not be directly used.

Value

Returns a `tibble` with the additional logical column `suffix_match_species_within_genus`, indicating whether the specific epithet was successfully matched within the matched genus (TRUE) or not (FALSE).

Examples

```
# substitute endings c('um$|i$|is$|us$|ae$') with 'a' of specific epithet
iucn_modified<- iucn %>%
  dplyr::mutate(Orig.Species = stringr::str_replace(Orig.Species, 'um$|i$|is$|us$|ae$', 'a'))
iucn_modified %>%
   dplyr::mutate(Matched.Genus = Orig.Genus) %>%
   suffix_match_species_within_genus(backbone = c('BGCI', 'WFO'))
```

---

summarize_output *Summarizes the output of the treemendous pipeline*

Description

Summarizes the output of the treemendous pipeline

Usage

```
summarize_output(df)
```

Arguments

df	: `tibble` being the output of matching()/sequential_matching()/enforce_matching() and optionally resolve_synonyms().

Value

Returns a `list` containing summary information about the matched species names and if provided also the resolved species names.

Examples

```
iucn %>% matching() %>% resolve_synonyms() %>% summarize_output()
```

translate_trees *Translate species names according to a custom target database.*

Description

The function is essentially a wrapper around the functions `matching()` and `enforce_matching()`. Species names from `df` are first directly matched to `target` by calling `matching(df, backbone = 'CUSTOM', target_df = target)`. Subsequently, the function calls `enforce_matching(df, backbone = 'CUSTOM', target_df = target)` to increase the number of translated species.

Usage

```
translate_trees(df, target, max_iter = 3)
```

Arguments

df	`tibble` with species that the user wants to translate into the species names of target. Species binomial split into the columns `Genus` and `Species`
target	`tibble` with a new custom target database. Species binomial split into the columns `Genus` and `Species`
max_iter	parameter which is passed to `enforce_matching()` and controls the maximum depth for matches.

Value

Returns a `tibble` with the species names of the input `df` in `Orig.Genus`, `Orig.Species`, and the translated names in `Matched.Genus` and `Matched.Species`. Process information from calling matching() and enforce_matching() is added to the output.

Examples

```
translate_trees(df = iucn, target = fia)
```

Treemendous.Trees *Database used by Treemendous to standardize Species Names*

Description

A dataset containing tree species assembled from four different publicly available datasets:

- **BGCI** (Botanical Gardens Conservation Internation): *GlobalTreeSearch*, Version `1.7` (April, 2023), Source
- **WFO** (World Flora Online): *Taxonomic Backbone*, Version `v.2023.06` (June, 2023), Source
- **WCVP** (World Checklist of Vascular Plants): Version `v9` (June, 2022), Source
- **GBIF** (Global Biodiversity Information Facility): Version (December, 2022), Source

Treemendous matches and resolves synonyms according to the dataset `Treemendous.Trees`, allowing the user always to specify a subset of the backbones if desired.

Usage

```
Treemendous.Trees
```

Format

A data frame with 401482 species and 38 variables:

Genus Genus name of species binomial

Species Specific epithet of species binomial

BGCI Boolean indicator whether this species was present in the `BGCI` backbone

WFO Boolean indicator whether this species was present in the `WFO` backbone

WCVP Boolean indicator whether this species was present in the `WCVP` backbone

GBIF Boolean indicator whether this species was present in the `GBIF` backbone

BGCI_Authors Information about authors based on `BGCI`

WFO_ID Unique ID in `WFO`

WFO_accepted_ID Unique ID of accepted species in `WFO`

WFO_Status Status according to `WFO`: e.g. Synonym, Accepted

WFO_Authors Information about authors based on `WFO`

WFO_Rank Taxonomic rank the species: one of `c('Species, Subspecies, Variety, Form)`

WFO_Family Taxonomical family as specified by `WFO`

WFO_Infraspecific Infraspecific epithet of the corresponding entry in `WFO`

WFO_infraspecific_ambiguity Boolean indicating whether multiple entries with identical latin binomials were present in the original **WFO** database, which would be resolved to different latin binomials in `resolve_synonyms()`. Specifically, the flag indicates when two or more entries at infraspecific levels would be resolved to different latin binomials. For more details, see `highlight_flags()`.

WFO_authorship_ambiguity Boolean indicating whether multiple entries with identical latin binomials were present in the original **WFO** database, which would be resolved to different latin binomials in `resolve_synonyms()`. Specifically, the flag indicates when Two or more entries at rank `Species` with different authorship would be resolved to different latin binomials. For more details, see `highlight_flags()`.

WFO_infraspecific_link Boolean indicator whether `WCVP_accepted_ID` was relinked to another entry in `Treemendous.Trees` with the same latin binomial. This was necessary because our database design only allowed for one entry for every unique latin binomial.

WCVP_ID Unique ID in `WCVP`

WCVP_accepted_ID Unique ID of accepted species in `WCVP`

WCVP_Status Status according to `WCVP`: e.g. Synonym, Accepted

WCVP_Authors Information about authors based on `WCVP`

WCVP_Rank Taxonomic rank the species: one of `c('Species, Subspecies, Variety, Form)`

WCVP_Family Taxonomical family as specified by `WCVP`

WCVP_Infraspecific Infraspecific epithet of the corresponding entry in `WCVP`

WCVP_infraspecific_ambiguity See `WCVP_infraspecific_ambiguity` above.

WCVP_authorship_ambiguity See `WCVP_authorship_ambiguity` above.

WCVP_infraspecific_link See `WCVP_infraspecific_link` above.

GBIF_ID Unique ID in GBIF

GBIF_accepted_ID Unique ID of accepted species in GBIF

GBIF_Status Status according to GBIF: e.g. Synonym, Accepted

GBIF_Authors Information about authors based on GBIF

GBIF_Rank Taxonomic rank the species: one of c('Species, Subspecies, Variety, Form)

GBIF_Family Taxonomical family as specified by GBIF

GBIF_Infraspecific Infraspecific epithet of the corresponding entry in GBIF

GBIF_infraspecific_ambiguity See GBIF_infraspecific_ambiguity above.

GBIF_authorship_ambiguity See GBIF_authorship_ambiguity above.

GBIF_infraspecific_link See GBIF_infraspecific_link above.

ID_merged Unique ID assigned to each species in Treemendous.Trees. Note that these ID's are currently not ensured to be consistent between subsequent versions of the package.

Details

See the publication accompanying the *Treemendous* package for more details. TODO: insert link to publication / pre-print.

The code for how the backbones were curated and merged is available in the package source code under data-raw/Treemendous-Trees.R.

Although all information about the taxonomic family of species (WFO_Family, WCVP_Family, GBIF_Family), as well as the scientific authorship (WFO_Authors, WCVP_Authors, GBIF_Authors, BGCI_Authors), is not used by the functionality of the package so far, we decided to keep the information out of the following reasons. First, it allows a user to further investigate matched names and allows for manual a assessment of whether a match was reasonable or not. Second, the unused information is likely to be used for future functionalities of the *Treemendous* package. For instance, we plan to let *Treemendous* interact with the *V.PhyloMaker2* package to get species phylogenies, a common analysis performed in ecological research. *V.PhyloMaker2* requires the user to input the taxonomic family, which could be resolved using the information about taxonomic families in Treemendous.Trees. Further, if a user has access to information about the scientific authorship, future versions might consider this in cases of ambiguous matches, and could help resolving these.

Source

https://github.com/speckerf/treemendous

Index

∗ **datasets**
fia, 5
iucn, 9
Treemendous.Trees, 14

direct_match, 2
direct_match(), *10*
direct_match_species_within_genus, 3
direct_match_species_within_genus(), *10*

enforce_matching, 3
enforce_matching(), *14*

fia, 5
fuzzy_match_genus, 5
fuzzy_match_genus(), *10*
fuzzy_match_species_within_genus, 6
fuzzy_match_species_within_genus(), *10*

genus_match, 7
genus_match(), *10*

highlight_flags, 7
highlight_flags(), *15*

iucn, 9

matching, 10
matching(), *4*, *10*, *14*

resolve_synonyms, 11

sequential_matching, 12
suffix_match_species_within_genus, 12
suffix_match_species_within_genus(), *10*
summarize_output, 13

translate_trees, 14
Treemendous.Trees, 14
